# Mechanical stimulation controls osteoclast function through the regulation of Ca^2+^-activated Cl^−^ channel Anoctamin 1

**DOI:** 10.1038/s42003-023-04806-1

**Published:** 2023-04-13

**Authors:** Weijia Sun, Yuheng Li, Jianwei Li, Yingjun Tan, Xinxin Yuan, Haoye Meng, Jianting Ye, Guohui Zhong, XiaoYan Jin, Zizhong Liu, Ruikai Du, Wenjuan Xing, Dingsheng Zhao, Jinping Song, Youyou Li, Junjie Pan, Yunzhang Zhao, Qi Li, Aiyuan Wang, Shukuan Ling, Rongji Dai, Yingxian Li

**Affiliations:** 1 Beijing Key Laboratory for Separation and Analysis in Biomedicine and Pharmaceuticals, Beijing Institute of Technology, Beijing, China; 2 State Key Laboratory of Space Medicine Fundamentals and Application, China Astronaut Research and Training Center, Beijing, China; 3 Institute of Orthopaedics, Chinese PLA General Hospital, Beijing, China; 4 Oujiang Laboratory (Zhejiang Lab for Regenerative Medicine, Vision and Brain Health), Wenzhou, Zhejiang China

**Keywords:** Molecular biology, Cell biology

## Abstract

Mechanical force loading is essential for maintaining bone homeostasis, and unloading exposure can lead to bone loss. Osteoclasts are the only bone resorbing cells and play a crucial role in bone remodeling. The molecular mechanisms underlying mechanical stimulation-induced changes in osteoclast function remain to be fully elucidated. Our previous research found Ca^2+^-activated Cl^−^ channel Anoctamin 1 (Ano1) was an essential regulator for osteoclast function. Here, we report that Ano1 mediates osteoclast responses to mechanical stimulation. In vitro, osteoclast activities are obviously affected by mechanical stress, which is accompanied by the changes of Ano1 levels, intracellular Cl^−^ concentration and Ca^2+^ downstream signaling. *Ano1* knockout or calcium binding mutants blunts the response of osteoclast to mechanical stimulation. In vivo, *Ano1* knockout in osteoclast blunts loading induced osteoclast inhibition and unloading induced bone loss and. These results demonstrate that Ano1 plays an important role in mechanical stimulation induced osteoclast activity changes.

## Abstract (web summary)

An essential role of the Ca2+-activated Cl^−^ channel, Anoctamin 1, is identified in mediating the response of osteoclasts to mechanical stimulation. Ano1 knockout in osteoclasts inhibits unloading- induced osteoclast activation and unloading-induced bone loss.

## Introduction

Bone remodeling is highly dependent on mechanical stimulation, as evidenced by increased bone mass in athletes and dramatic bone loss under conditions of longtime bed rest^1^ or microgravity during spaceflight^2^. Mechanical stress enhances bone mass primarily through increasing osteoblast number, promoting its activity, and up-regulating its recruitment, thereby stimulating bone formation^3,4^. Conversely, the absence of mechanical stimulation reduces bone mass by inhibiting bone formation^5^ and promoting osteoclastogenesis and sunbsequent bone resorption^6^. Traditionally, osteoblasts and osteocytes have been thought to be the cells responsible for sensing mechanical stimuli, and their roles in bone metabolism have been extensively documented^7,8^. Nevertheless, the effects of mechanical stimuli on osteoclasts were scarcely reported and the mechanism of osteoclast response to mechanical stress remains to be fully understood. To effectively utilize the mechanical signals in the clinic as a non-drug-based intervention for osteoporosis, it is essential to identify the components of the mechanical challenge that are critical for the bone remodeling.

Mechanical stimuli represent important environmental cues that living organisms have to sense and cope with^5,9^. Mechanical force sensing is conserved across all domains of life, with a variety of proteins involved in sensing and responding to mechanical forces^10–12^. Among them, mechanosensitive ion channels are activated directly by mechanical forces applied to the cells^13,14^. Cells convert mechanical stimuli into electrical or chemical signals via these mechanosensitive ion channels, which open and close in response to mechanical stimuli^15,16^. Our previous finding revealed that mechanosensitive Piezo1 channel in osteoblast is required for bone formation, as it functions as a key transducer of mechanosensitive in osteoblasts^8^. However, the mechanosensitive ion channels in osteoclasts remain less studied.

Anoctamin-1 (ANO1), also known as TMEM16A, was identified as calcium-activated chloride channel. ANO1 plays an important role in a variety of cells, including the control of the excitability of smooth muscle cells^17,18^ and neurons^19^, fluid secretion by epithelial cells^20^, acute pain sensation^21^, olfactory transduction^22^, cancer cell proliferation, survival and migration^23,24^. Our previous study showed that ANO1 is an essential regulator of osteoclast function, and its channel activity contributes to its interaction with RANK, which promotes RANKL-induced downstream signaling pathways^25^.

The structure of cryo-EM of Ano1 reveals that each subunit includes ten transmembrane (TM) segments, with TM3–8 lining one ion-conductive path. Bioinformatics analyses based on strategies for identifying distant phylogenetic relationships suggest Ano1 is evolutionarily related to the mechanosensitive channels OSCA (hyperosmolality-gated calcium-permeable channel)^26^. Moreover, given the features of the cryo-EM map and the structural similarities between the mechanosensitive OSCA channels and the ANO1 channel, we propose that ANO family proteins possess the same mechanosensitive characteristics as the mechanosensitive OSCA ion channels.

In this study, we found that mechanical stimulation leads to changes in osteoclasts function and Ano1 plays an essential role in this process. Mechanical stimulation induced by fluid shear stress (FSS) or hypergravity (HG) inhibited osteoclast activity, which was accompanied with a decrease in Ano1 levels. Conversely, mechanical unloading under simulated microgravity condition (MG) result in an increase in Ano1 levels and subsequently promotes osteoclast activity. ANO1 deficiency in osteoclast reduces its sensitivity to mechanical stimulation. Mechanistically, ANO1 channel activity was modified by mechanical signaling, leading to changes in intracellular Cl^−^ concentration and calcium-mediated pathways. Ano1 knockout in osteoclast significantly inhibits unloading- induced osteoclast activation and alleviates unloading-induced bone loss. These results demonstrate that Ano1 is an intrinsic factor that enables osteoclasts to respond to mechanical stimulation.

## Results

### The effect of different mechanical stimulation on osteoclastogenesis and the changes of Ano1 levels during this process

**Fig. 1:**
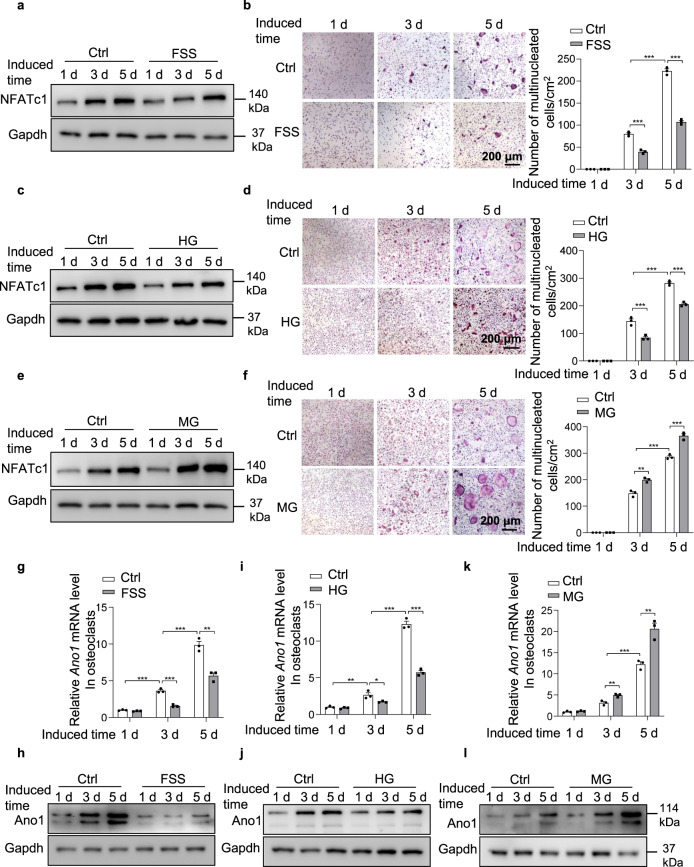
The osteoclast activity alteration induced by mechanical stimulation is accompanied with the changes of Ano1 expression. **a** Western blot analysis of NFATc1 protein level during osteoclast differentiation under fluid shear stress (FSS) treatment for 1 day, 3 days or 5 days. **b** Representative images of TRAP staining during osteoclast differentiation under FSS treatment for 1 day, 3 days or 5 days (left). Scale bar, 200 μm. Quantification of the number of multinucleated cells per cm^2^ (right). (*n* = 0–231, from three independent experiments). **c** Western blot analysis of NFATc1 protein level during osteoclast differentiation under hypergravity (HG) treatmentfor 1 day, 3 days or 5 days. **d** Representative images of TRAP staining during osteoclast differentiation under HG treatment for 1 day, 3 days or 5 days (left). Scale bar, 200 μm. Quantification of the number of multinucleated cells per cm^2^ (right). (*n* = 0–291, from three independent experiments). **e** Western blot analysis of NFATc1 protein level during osteoclast differentiation under microgravity (MG) treatment for 1 day, 3 days or 5 days. **f** Representative images of TRAP staining during osteoclast differentiation under MG treatment for 1 day, 3 days or 5 days (left). Scale bar, 200 μm. Quantification of the number of multinucleated cells per cm^2^ (right). (*n* = 0–377, from three independent experiments). **g** QRT-PCR analysis of Ano1 mRNA level during osteoclast differentiation under FSS treatment for 1 day, 3 days or 5 days. (*n* = 3 independent experiments). **h** Western blot analysis of Ano1 protein level during osteoclast differentiation under FSS treatment for 1 day, 3 days or 5 days. **i** QRT-PCR analysis of Ano1 mRNA level during osteoclast differentiation under HG treatment for 1 day, 3 days or 5 days. (*n* = 3 independent experiments). **j** Western blot analysis of Ano1 protein level during osteoclast differentiation under HG treatment for 1 day, 3 days or 5 days. **k** QRT-PCR analysis of Ano1 mRNA level during osteoclast differentiation under MG treatment for 1 day, 3 days or 5 days. (*n* = 3 independent experiments). **l** Western blot analysis of Ano1 protein level during osteoclast differentiation under MG treatment for 1 day, 3 days or 5 days. All data are the mean ± s.e.m. Statistical analysis with more than two groups was performed with two-way analysis of variance (ANOVA) with Šídák post-hoc test to determine group differences. *p < 0.05, **p < 0.01, ***p < 0.001.

To examine the effect of mechanical stimulation on osteoclast activity, we utilized the following three mechanical forces, fluid shear stress (FSS), hypergravity (HG), and simulated microgravity (MG), to stimulate the osteoclasts. Twelve dyn/cm^2^ FSS and 4 g HG treatment were used to investigate the effect of mechanical loading and the simulated microgravity system was used to investigate the effect of mechanical unloading on osteoclast differentiation and activities (Supplementary Fig. 1a–c). Mouse bone marrow-derived macrophages (BMMs) were cultured with M-CSF (10 ng/ml) alone for 1 day, and followed with M-CSF (30 ng/ml) and RANKL (50 ng/ml) for 1 day, 3 days and 5 days. Cells were treated with FSS, HG and MG in the whole process. We examined the expression of the marker genes of each stage during osteoclasts differentiation^27–29^. When cells were treated with FSS on day 1, there was no change in the expression of *integrin subunit alpha X* (*Itgax*) and *Cd74*, marker genes of the early stage of osteoclasts differentiation (Supplementary Fig. 2a). On day 3, the expression of *dendrocyte expressed seven transmembrane protei*n (*Dcstamp*) and *ATPase H*^*+*^ *transporting V0 subunit d2* (*Atp6v0d2*), marker genes of preosteoclast, was suppressed (Supplementary Fig. 2b). On day 5, the expression of *nuclear factor of activated T cell 1 (NFATc1), acid phosphatase 5 (Acp5), cathepsin K (Ctsk)* and *matrix metalloprotein 9 (Mmp9)*, marker genes of osteoclast activity, was significantly decreased (Supplementary Fig. 2c). The protein level of NFATc1 was increased during osteoclast differentiation, and FSS inhibited NFATc1 protein level (Fig. 1a). The number of TRAP^+^ cells were decreased in FSS treated osteoclasts than that in control osteoclasts (Fig. 1b). Similar results were observed in osteoclasts after treatment with HG, which didn’t affect the expression of Itgax and Cd74, down-regulated the expression of *Dcstamp, Atp6v0d2, NFATc1*, *Acp5*, *Ctsk* and *Mmp9*, inhibited NFATc1 protein level and decreased the number of TRAP^+^ cells (Supplementary Fig. 2d–f, Fig. 1c, d). Microgravity obviously enhanced the differentiation and activities of osteoclasts, the expression of genes in the early stage of osteoclast differentiation remained unchanged (Supplementary Fig. 2g), while that of preosteoclast and osteoclast genes was significantly higher under treatment with MG than that in control cells (Supplementary Fig. 2h, i). The protein level of NFATc1 and the number of TRAP^+^ cells were significantly increased in MG treated osteoclasts than that in control osteoclasts (Fig. 1e, f).

These results suggested that mechanical force affected osteoclast activity. Our previous study found Ano1 plays an essential role in osteoclast activity. The features of Ano1 cryo-EM map and the structural similarity of Ano1 with mechanosensitive OSCA prompted us to test whether Ano1 was involved in osteoclast response to mechanical stimulation. Under FSS or HG treatment, Ano1 levels in osteoclasts were significantly decreased (Fig. 1g–j). When subjected to MG treatment, Ano1 levels were significantly increased in osteoclasts (Fig. 1k, l). These results suggested that Ano1 is associated with the changes of osteoclast activities induced by mechanical stimulation.

### The role of Ano1 in mechanical loading-induced reduction of osteoclast function

**Fig. 2:**
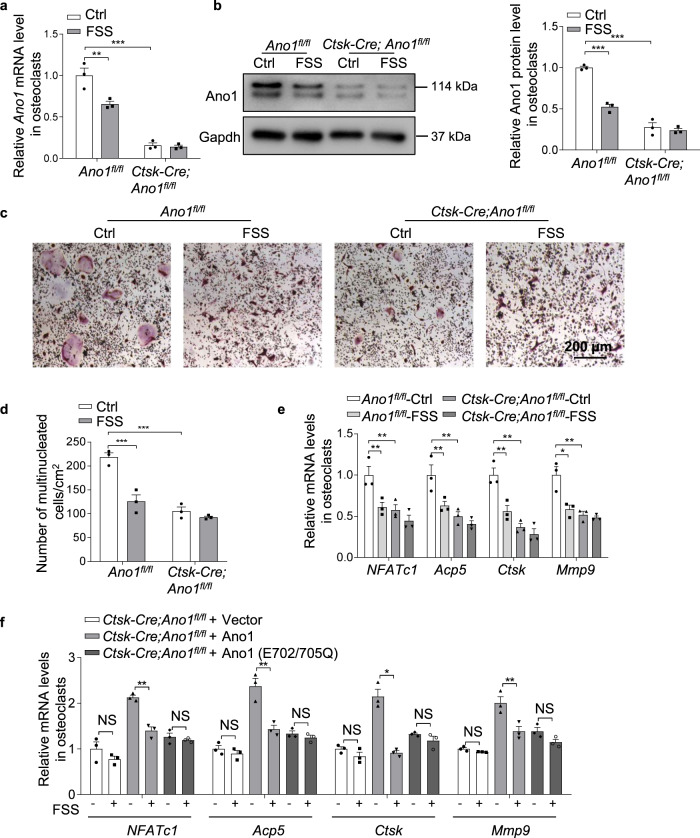
Ano1 knock out blunts the inhibition of fluid shear stress on osteoclast activity. **a** QRT-PCR analysis of *Ano1* mRNA level in *Ano1*^*fl/fl*^ and *Ctsk-Cre;Ano1*^*fl/fl*^ osteoclasts after treatment with Ctrl or FSS (12 dyn/cm^2^, 30 min/day) for 2 days. (*n* = 3 independent experiments). **b** Western blot analysis of Ano1 protein level in *Ano1*^*fl/fl*^ and *Ctsk-Cre;Ano1*^*fl/fl*^ osteoclasts after treatment with Ctrl or FSS (left). The quantification of Ano1 protein level in osteoclasts (right). (*n* = 3 independent experiments). **c** Representative images of TRAP staining in *Ano1*^*fl/fl*^ and *Ctsk-Cre;Ano1*^*fl/fl*^ osteoclasts after treatment with Ctrl or FSS (12 dyn/cm^2^, 30 min/day) for 2 days. Scale bar, 200 μm. **d** Quantification of the number of multinucleated cells per cm^2^. (*n* = 89–231, from three independent experiments). **e** QRT-PCR analysis of *NFATc1*, *Acp5*, *Ctsk* and *Mmp9* mRNA levels in osteoclasts *Ano1*^*fl/fl*^ and *Ctsk-Cre;Ano1*^*fl/fl*^ osteoclasts after treatment with Ctrl or FSS for 2 days. (*n* = 3 independent experiments). **f** QRT-PCR analysis of *NFATc1*, *Acp5*, *Ctsk* and *Mmp9* mRNA levels in osteoclasts isolated from *Ctsk-Cre;Ano1*^*fl/fl*^ mice. Osteoclasts were transfected with NC, WT *Ano1* or mutant *Ano1* (E702/705Q) and treated with or without FSS for 2 days. (*n* = 3 independent experiments).All data are the mean ± s.e.m. Statistical analysis with more than two groups was performed with two-way analysis of variance (ANOVA) with Šídák post-hoc test to determine group differences. *p < 0.05, **p < 0.01, ***p < 0.001.

**Fig. 3:**
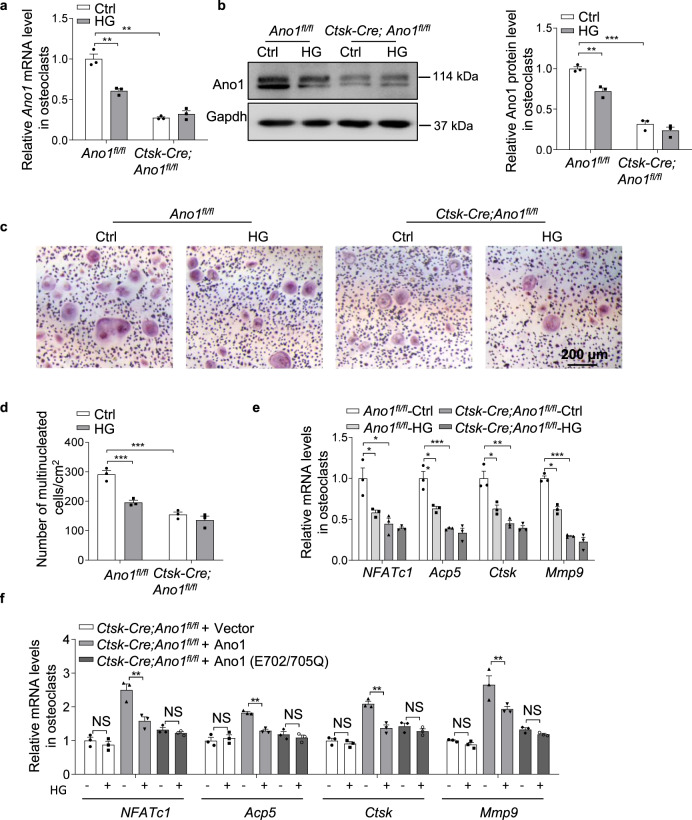
Ano1 knock out blunts the inhibition of hypergravity on osteoclast activity. **a** QRT-PCR analysis of *Ano1* mRNA level in *Ano1*^*fl/fl*^ and *Ctsk-Cre;Ano1*^*fl/fl*^ osteoclasts after treatment with Ctrl or HG (4 g) for 2 days. (*n* = 3 independent experiments). **b** Western blot analysis of Ano1 protein level in *Ano1*^*fl/fl*^ and *Ctsk-Cre;Ano1*^*fl/fl*^ osteoclasts after treatment with Ctrl or HG (left). The quantification of Ano1 protein level in osteoclasts (right). (*n* = 3 independent experiments). **c** Representative images of TRAP staining in *Ano1*^*fl/fl*^ and *Ctsk-Cre;Ano1*^*fl/fl*^ osteoclasts after treatment with Ctrl or HG (4 g) for 2 days. Scale bar, 200 μm. **d** Quantification of the number of multinucleated cells per cm^2^. (*n* = 109–312, from three independent experiments). **e** QRT-PCR analysis of *NFATc1*, *Acp5*, *Ctsk* and *Mmp9* mRNA levels in *Ano1*^*fl/fl*^ and *Ctsk-Cre;Ano1*^*fl/fl*^ osteoclasts after treatment with Ctrl or HG. (*n* = 3 independent experiments). **f** QRT-PCR analysis of *NFATc1*, *Acp5*, *Ctsk* and *Mmp9* mRNA levels in osteoclasts isolated from *Ctsk-Cre;Ano1*^*fl/fl*^ mice. Osteoclasts were transfected with NC, WT *Ano1* or mutant *Ano1* (E702/705Q) and treated with or without HG for 2 days. (*n* = 3 independent experiments).All data are the mean ± s.e.m. Statistical analysis with more than two groups was performed with two-way analysis of variance (ANOVA) with Šídák post-hoc test to determine group differences. *p < 0.05, **p < 0.01, ***p < 0.001.

To determine whether Ano1 mediated the inhibition role of mechanical-loading in osteoclast, we isolated osteoclast from osteoclast-specific conditional *Ano1* knockout (*Ctsk-Cre;Ano1*^*fl/fl*^) mice. The results showed that the activities of *Ctsk-Cre;Ano1*^*fl/fl*^ osteoclasts were significantly lower than that in *Ano1*^*fl/fl*^ osteoclasts (Fig. 2a–e). We subjected the *Ano1*^*fl/fl*^ and *Ctsk-Cre;Ano1*^*fl/fl*^ osteoclasts to FSS for 30 min per day for 2 days and HG for 2 days, respectively. The *Ano1* mRNA level was decreased by 34.53% and the protein level of Ano1 was decreased by 47.8% after FSS treatment in the *Ano1*^*fl/fl*^ osteoclasts, but no significant changes in the *Ctsk-Cre;Ano1*^*fl/fl*^ osteoclasts (Fig. 2a, b). FSS treatment led to 40.7% reduction in the number of TRAP^+^ multinucleated cells and significantly decrease the expression of *NFATc1*, *Acp5*, *Ctsk* and *Mmp9* in the *Ano1*^*fl/fl*^ osteoclasts (Fig. 2c–e). It is known that the conserved amino acid residues E702 and E705 are two key sites for Ca^2+^-dependent channel activation, which are important for Ano1 activity. To determine whether the calcium binding site plays a key role in Ano1-mediated mechanical stimulation, *Ctsk-Cre;Ano1*^*fl/fl*^ osteoclasts were transfected with vector, wild-type Ano1 (Ano1) or mutant Ano1 (E702/E705Q), and then treated with FSS. We found that *Ctsk-Cre;Ano1*^*fl/fl*^ osteoclasts did not respond to FSS, wild-type Ano1 can rescue the response of *Ctsk-Cre;Ano1*^*fl/fl*^ osteoclasts to FSS, but not Ano1 with Ca^2+^ binding sites mutants (Fig. 2f). Similarly, HG treatment resulted in decrease both in Ano1 mRNA and protein levels (Fig. 3a, b), and a 22.5% reduction in osteoclast number in the *Ano1*^*fl/fl*^ osteoclasts, but not in the *Ctsk-Cre;Ano1*^*fl/fl*^ osteoclasts (Fig. 3c, d). Accordingly, the expressions of *NFATc1*, *Acp5*, *Ctsk* and *Mmp9* were also inhibited in the *Ano1*^*fl/fl*^ osteoclasts, but not in the *Ctsk-Cre;Ano1*^*fl/fl*^ osteoclasts (Fig. 3e). *Ctsk-Cre;Ano1*^*fl/fl*^ osteoclasts also did not respond to HG, wild-type Ano1 can rescue the response of *Ctsk-Cre;Ano1*^*fl/fl*^ osteoclasts to HG, but not Ano1 with Ca^2+^ binding sites mutants (Fig. 3f). These data suggest that Ano1 mediated mechanical loading induced the reduction of osteoclast activity.

To determine whether Ano1 in osteoclast involved in the response to mechanical loading in vivo, we loaded the left tibia of 16-week-old female *Ctsk-Cre;Ano1*^*fl/fl*^ mice and *Ano1*^*fl/fl*^ mice with +1200 μɛ peak strain at the midshaft^30^. TRAP staining showed that mechanical loading for 2 weeks reduced Oc.S/BS and N.Oc/B.Pm of the tibia in *Ano1*^*fl/fl*^ mice but not in *Ctsk-Cre;Ano1*^*fl/fl*^ mice (Supplementary Fig. 3a, b).The expression of *NFATc1*, *Acp5*, *Ctsk* and *Mmp9* were specifically decreased in bone of *Ano1*^*fl/fl*^ mice treated with mechanical loading, but not in *Ctsk-Cre;Ano1*^*fl/fl*^ mice (Supplementary Fig. 3c, d). These results suggest that Ano1 in osteoclasts plays an important role in the response of the bone to mechanical loading.

### The role of Ano1 in mechanical unloading-induced enhancement of osteoclast function

**Fig. 4:**
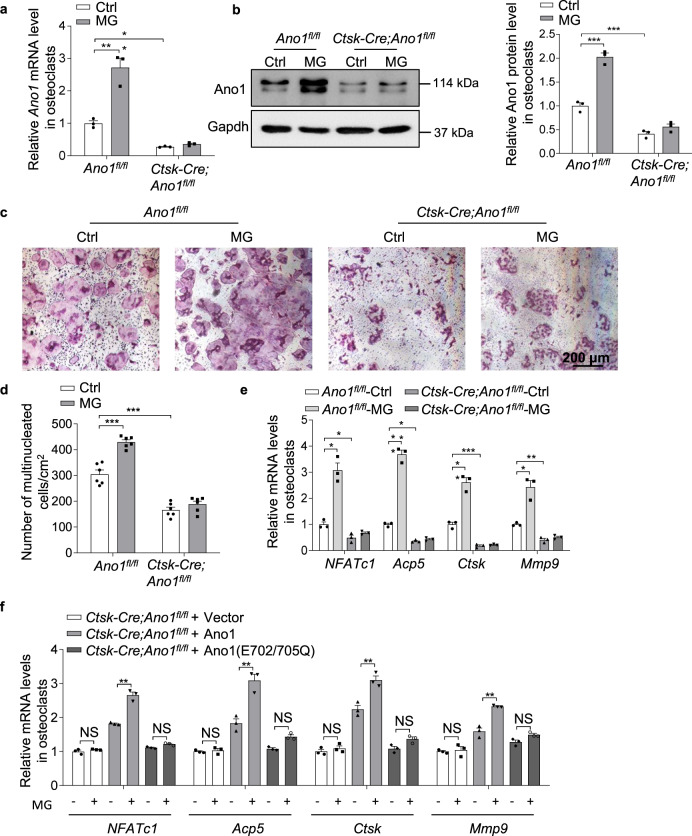
Ano1 knock out alleviates the enhancement of mechanical unloading on osteoclast activity. **a** QRT-PCR analysis of *Ano1* mRNA level in *Ano1*^*fl/fl*^ and *Ctsk-Cre;Ano1*^*fl/fl*^ osteoclasts after treatment with Ctrl or MG for 2 days. (*n* = 3 independent experiments). **b** Western blot analysis of Ano1 protein level in *Ano1*^*fl/fl*^ and *Ctsk-Cre;Ano1*^*fl/fl*^ osteoclasts after treatment with Ctrl or MG for 2 days (left). The quantification of Ano1 protein level in osteoclasts (right). (*n* = 3 independent experiments). **c** Representative images of TRAP staining in *Ano1*^*fl/fl*^ and *Ctsk-Cre;Ano1*^*fl/fl*^ osteoclasts after treatment with Ctrl or MG for 2 days. Scale bar, 200 μm. **d** Quantification of the number of multinucleated cells per cm^2^. (*n* = 130–453, from six independent experiments). **e** QRT-PCR analysis of *NFATc1*, *Acp5*, *Ctsk* and *Mmp9* mRNA levels in osteoclasts *Ano1*^*fl/fl*^ and *Ctsk-Cre;Ano1*^*fl/fl*^ osteoclasts after treatment with Ctrl or MG. (*n* = 3 independent experiments). **f** QRT-PCR analysis of *NFATc1*, *Acp5*, *Ctsk* and *Mmp9* mRNA levels in osteoclasts isolated from *Ctsk-Cre;Ano1*^*fl/fl*^ mice. Osteoclasts were transfected with NC, WT *Ano1* or mutant *Ano1* (E702/705Q) and treated with or without MG for 2 days. (*n* = 3 independent experiments). All data are the mean ± s.e.m. Statistical analysis with more than two groups was performed with two-way analysis of variance (ANOVA) with Šídák post-hoc test to determine group differences. *p < 0.05, **p < 0.01, ***p < 0.001.

To determine whether Ano1 mediated the response of osteoclast to mechanical unloading, osteoclasts from *Ano1*^*fl/fl*^ and *Ctsk-Cre;Ano1*^*fl/fl*^ mice were subjected to unloading treatment. After treatment with MG for 2 days, the mRNA and protein levels of Ano1 were significantly increased by 2.7 folds and 2 folds in the *Ano1*^*fl/fl*^ osteoclasts, respectively, but not in the *Ctsk-Cre;Ano1*^*fl/fl*^ osteoclasts (Fig. 4a, b). MG treatment caused 2-fold increase in the number of TRAP^+^ multinucleated cells and significantly increased the expression of osteoclast marker genes in the *Ano1*^*fl/fl*^ osteoclasts, but not in the *Ctsk-Cre;Ano1*^*fl/fl*^ osteoclasts (Fig. 4c–e). Similarly, wild-type Ano1 can rescue the response of *Ctsk-Cre;Ano1*^*fl/fl*^ osteoclasts to MG, but not Ano1 with Ca^2+^ binding sites mutants (Fig. 4f).These data suggest that Ano1 knock out in osteoclast eliminates mechanical unloading induced the enhancement of osteoclast activity.

### The changes of intracellular Cl^−^ under mechanical stimulation and the role of Ano1 in this process

**Fig. 5:**
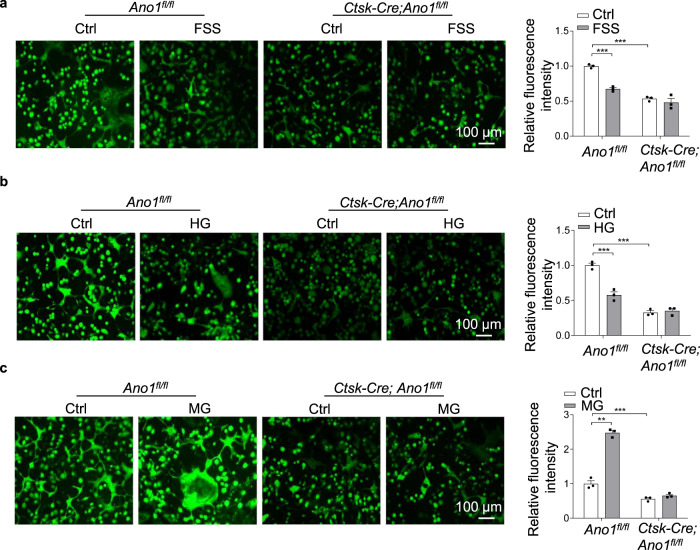
The mechanical stimulation can induce the changes of intracellular chloride through Ano1 in osteoclast. Measurement of intracellular chloride concentration in osteoclasts. All the cells were stained with 5 mM MQAE. **a** Representative fluorescent images of *Ano1*^*fl/fl*^ and *Ctsk-Cre;Ano1*^*fl/fl*^ osteoclasts after treatment with Ctrl or FSS (12 dyn/cm^2^, 30 min/day) for 2 days (left). Scale bar, 100 μm. The relative fluorescence intensity of osteoclasts (right). (*n* = 3 independent experiments). **b** Representative fluorescent images of *Ano1*^*fl/fl*^ and *Ctsk-Cre;Ano1*^*fl/fl*^ osteoclasts after treatment with Ctrl or HG (4 g) for 2 days (left). Scale bar, 100 μm.The relative fluorescence intensity of osteoclasts (right). (*n* = 3 independent experiments). **c** Representative fluorescent images of *Ano1*^*fl/fl*^ and *Ctsk-Cre;Ano1*^*fl/fl*^ osteoclasts after treatment with Ctrl or MG for 2 days (left). Scale bar, 100 μm. The relative fluorescence intensity of osteoclasts (right). (*n* = 3 independent experiments). All data are the mean ± s.e.m. Statistical analysis with more than two groups was performed with two-way analysis of variance (ANOVA) with Šídák post-hoc test to determine group differences. **p < 0.01, ***p < 0.001.

Based on the characteristics of Ano1 as a chloride channel, we investigate the changes of cytoplasmic Cl^−^ level in osteoclast in response to mechanical stimulation. We used a Cl^−^ sensor (the fluorescent probe, MQAE), the fluorescence intensity of this sensor is inversely related to the intracellular Cl^−^ concentration, and a decrease in the fluorescence intensity correlates with an increase in the cytoplasmic Cl^−^ level^31^. Intriguingly, when subjected to the FSS and HG treatment, the fluorescence intensity of the Cl^−^ sensor was significantly reduced by 46.5% and 42.4% by an increase in the intracellular Cl^−^ concentration in the *Ano1*^*fl/fl*^ osteoclasts, but not in the *Ctsk-Cre;Ano1*^*fl/f*l^ osteoclasts (Fig. 5a, b). However, when subjected to the MG treatment, the fluorescence intensity of the Cl^−^ sensor was increased, but not in the *Ctsk-Cre;Ano1*^*fl/f*l^ osteoclasts (Fig. 5c). These results indicated the mechanical loading could increase intracellular Cl^−^ levels and unloading decrease intracellular Cl^−^ levels in osteoclast, which was mediated by Ano1.

### Ano1 mediates osteoclast response to mechanical stimulation through CaMKIV/ Creb/NFATc1 signaling

**Fig. 6:**
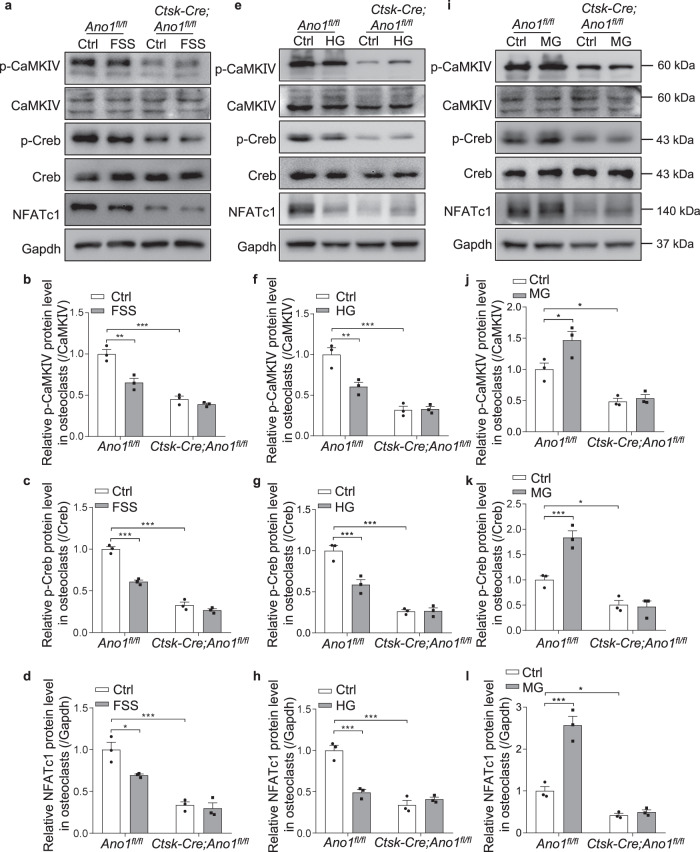
Ano1 regulates calcium activated signaling in response to mechanical stimulation in osteoclast. **a** Western blot analysis of p-CaMKIV, CaMKIV, p-Creb,Creb and NFATc1 protein levels in *Ano1*^*fl/fl*^ and *Ctsk-Cre;Ano1*^*fl/fl*^ osteoclasts with Ctrl or FSS (12 dyn/cm^2^, 30 min/day) treatment for 2 days. **b**–**d** The quantification of p-CaMKIV, p-Creb and NFATc1 protein levels in osteoclasts. The p-CaMKIV protein level was normalized to CaMKIV, the p-Creb protein level was normalized to Creb, and the NFATc1 protein level was normalized to Gapdh. (*n* = 3 independent experiments). **e** Western blot analysis of p-CaMKIV, CaMKIV, p-Creb,Creb and NFATc1 protein levels in *Ano1*^*fl/fl*^ and *Ctsk-Cre;Ano1*^*fl/fl*^ osteoclasts with Ctrl or HG (4 g) treatment for 2 days. **f**–**h** The quantification of p-CaMKIV, p-Creb and NFATc1 protein levels in osteoclasts. The p-CaMKIV protein level was normalized to CaMKIV, the p-Creb protein level was normalized to Creb, and the NFATc1 protein level was normalized to Gapdh. (*n* = 3 independent experiments). **i** Western blot analysis of p-CaMKIV, CaMKIV, p-Creb,Creb and NFATc1 protein levels in *Ano1*^*fl/fl*^ and *Ctsk-Cre;Ano1*^*fl/fl*^ osteoclasts with Ctrl or MG treatment for 2 days. **j**–**l** The quantification of p-CaMKIV, p-Creb and NFATc1 protein levels in osteoclasts. The p-CaMKIV protein level was normalized to CaMKIV, the p-Creb protein level was normalized to Creb, and the NFATc1 protein level was normalized to Gapdh. (*n* = 3 independent experiments). All data are the mean ± s.e.m. Statistical analysis was performed with two-way analysis of variance (ANOVA) with Šídák post-hoc test to determine group differences. *p < 0.05, **p < 0.01, ***p < 0.001.

Our previous study found Ano1 was required for RANKL-mediated CaMKIVCreb activation during osteoclastogenesis. To determine whether Ano1 mediates osteoclast response to mechanical stimulation through CaMKIV-Creb signaling pathway, we tested the effect of mechanical loading and unloading on the phosphorylation of CaMKIV and Creb. The results showed that the phosphorylation of CaMKIV and Creb were decreased, which was accompanied by downregulation of NFATc1 in the *Ano1*^*fl/fl*^ osteoclasts after treatment with FSS or HG, but there was no change in the *Ctsk-Cre;Ano1*^*fl/fl*^ osteoclasts (Fig. 6a–h). The phosphorylation of CaMKIV and Creb, and NFATc1 protein levels were all increased in the *Ano1*^*fl/fl*^ osteoclasts after treatment with MG, but there was no change in the *Ctsk-Cre;Ano1*^*fl/fl*^ osteoclasts between Ctrl and MG group (Fig. 6i–l). These results indicated that CaMKIV-Creb-NFATc1 signaling is involved in Ano1 mediated mechanical stimulation on osteoclast activity.

### *Ano1* knockout in osteoclast attenuates unloading induced bone loss

**Fig. 7:**
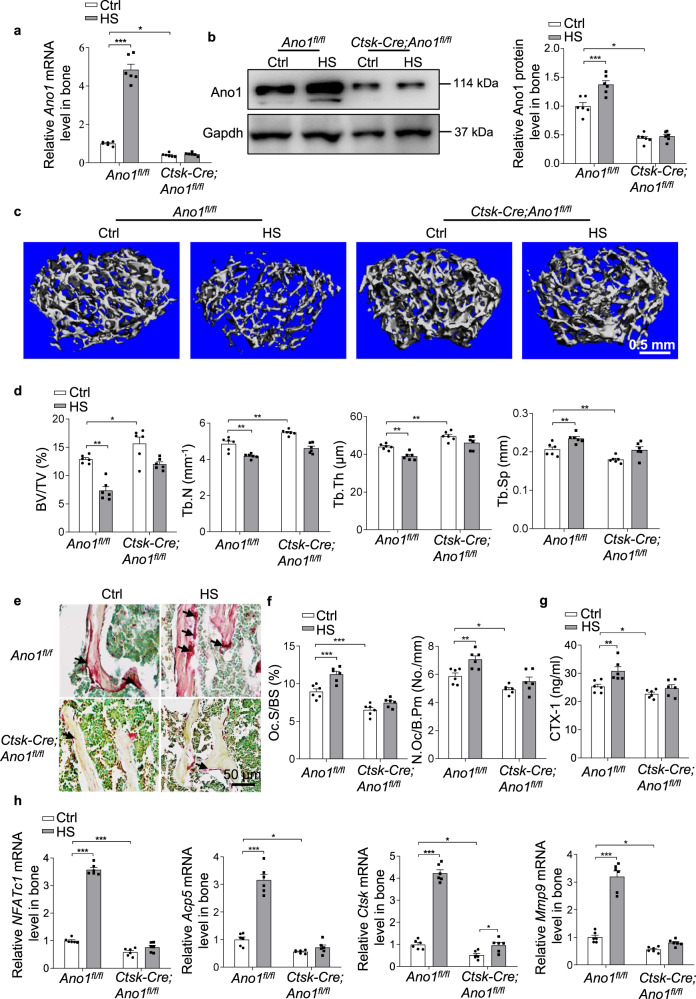
Osteoclast specific Ano1 knock out protects from unloading induced bone loss. **a**, **b** QRT-PCR analysis of *Ano1* mRNA level and western blot analysis of Ano1 protein level in bone tissues from *Ano1*^*fl/fl*^ and *Ctsk-Cre;Ano1*^*fl/fl*^ mice with Ctrl or hind limb suspension (HS) treatment. (*n* = 6 for each group). **c** Representative images showing three-dimensional trabecular architecture as determined by micro-CT reconstruction of the distal femurs from the groups of mice indicated. Scale bar, 0.5 mm. **d** Micro-CT measurements for bone volume per tissue volume (BV/TV), trabecular number (Tb.N), trabecular thickness (Tb.Th) and trabecular spacing (Tb.Sp) in the distal femurs from the groups of mice indicated. (*n* = 6 for each group). **e** Representative images of TRAP staining of the proximal tibia from the groups of mice indicated. Scale bar, 50 μm. **f** Histomorphometry analysis of the images for number of osteoclasts per bone perimeter (N.Oc/B.Pm) and osteoclast surface per bone surface (Oc.S/BS). (*n* = 6 for each group). **g** ELISA analysis of CTX-1 protein level in serum from the groups of mice indicated. (*n* = 6 for each group). **h** QRT-PCR analysis of *NFATc1*, *Acp5*, *Ctsk* and *Mmp9* mRNA levels in bone tissues collected from the groups of mice indicated. (*n* = 6 for each group). Statistical analysis with more than two groups was performed with two-way analysis of variance (ANOVA) with Šídák post-hoc test to determine group differences. *p < 0.05, **p < 0.01, ***p < 0.001.

To determine whether Ano1 mediates mechanical unloading induced bone loss, we used hindlimb suspension (HS) model to examine the hindlimb bone changes in response to the weight-bearing unloading. The three-month-old *Ano1*^*fl/fl*^ and *Ctsk-Cre;Ano1*^*fl/fl*^ mice were subjected to HS for 28 days. The mRNA and protein levels of Ano1 were significantly increased in bone tissues from *Ano1*^*fl/fl*^ mice subjected to HS (Fig. 7a, b). Micro-CT analysis showed that the trabecular bone mass and architecture related parameters, including the ratio of bone volume to tissue volume (BV/TV), trabecular number (Tb.N), and trabecular thickness (Tb.Th), were significantly reduced and the trabecular spacing (Tb.Sp) was accordingly increased in the *Ano1*^*fl/fl*^ mice after treatment with HS (Fig. 7c, d). *Ctsk-Cre;Ano1*^*fl/fl*^ mice subjected to HS displayed much higher trabecular bone mass, BV/TV, Tb.N and Tb.Th than that in *Ano1*^*fl/fl*^ mice subjected to HS. Accordingly, Tb.Sp was much lower in *Ctsk-Cre;Ano1*^*fl/fl*^ mice than that in the control mice (Fig. 7c, d). Next, we analyzed the corresponding changes in osteoclast function after HS treatment. TRAP staining showed obviously increased osteoclast activity in the *Ano1*^*fl/fl*^ mice subjected to HS. However, HS induced only a small increase in TRAP activity in *Ctsk-Cre;Ano1*^*fl/fl*^ mice, which was much weaker than that observed in *Ano1*^*fl/fl*^ mice (Fig. 7e). Osteoclast surface per bone surface (Oc.S/BS) and number of osteoclasts per bone perimeter (N.Oc/B.Pm) in the proximal tibia of *Ctsk-Cre;Ano1*^*fl/fl*^ mice subjected to HS were much lower than that in the *Ano1*^*fl/fl*^ mice (Fig. 7f). Accordingly, the level of CTX-1 showed significantly increased in serum from the *Ano1*^*fl/fl*^ mice, while with a little increase in *Ctsk-Cre;Ano1*^*fl/fl*^ mice (Fig. 7g). QRT-PCR analysis showed that *NFATc1*, *Acp5*, *Ctsk* and *Mmp9* mRNA levels were all significantly reduced in bone tissue from *Ctsk-Cre;Ano1*^*fl/fl*^ mice subjected to HS compared with the *Ano1*^*fl/fl*^ control mice subjected to HS (Fig. 7h). To verify the change of bone formation in *Ctsk-Cre;Ano1*^*fl/fl*^ mice and *Ano1*^*fl/fl*^ mice after HS treatment, we injected calcein intraperitoneally into mice on 10 days and 2 days before euthanasia to label new bone formation. After treatment with HS, the mineral apposition rate (MAR) and bone formation rate per bone surface (BFR/BS) in the tibias of *Ano1*^*fl/fl*^ mice decreased by 31% and 17%, respectively. Their levels in the tibias of *Ctsk-Cre;Ano1*^*fl/fl*^ mice after HS treatment decreased by 21% and 4% compared with Ctrl group, respectively (Supplementary Fig. 4a, b).

## Discussion

**Fig. 8:**
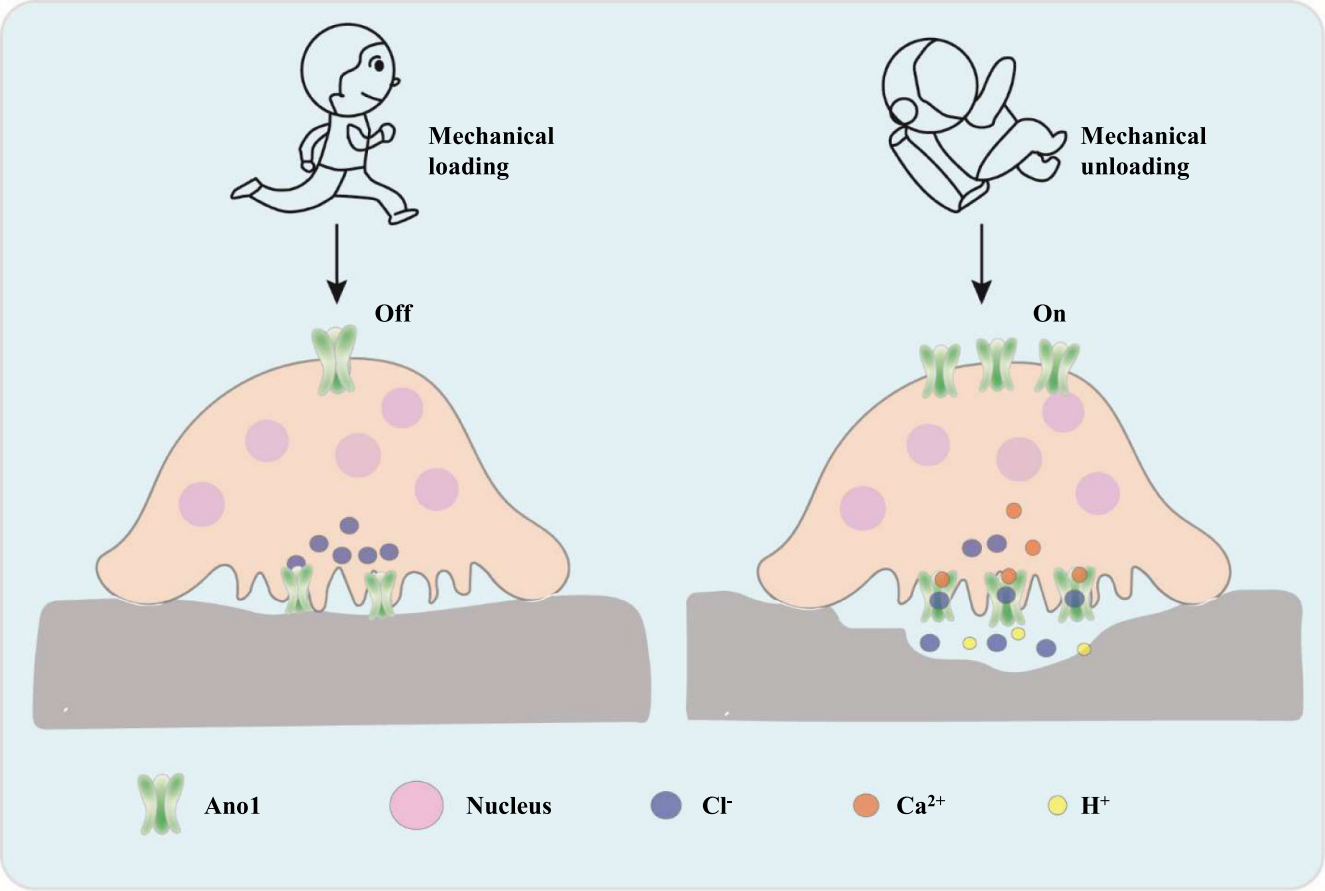
Schematic model depicting the role of Ano1 in the regulation of mechanical stimulation on osteoclas. Ano1 functions a mechanical sensor in osteoclast through its calcium regulated channel activities.

In this study, we demonstrate that Ano1is a previously unidentified mechanosensitive channel in osteoclasts. We found that osteoclast function is regulated by mechanical treatment, which is accompanied with the changes of Ano1 levels. Under 12 dyn/cm^2^ fluid shear stress or 4 g hypergravity conditions, osteoclast activity was inhibited and the expression of Ano1 was decreased. Conversely, simulated microgravity promotes osteoclast activity and increases the levels of Ano1. Here, we identified Ano1functions as an important regulator in osteoclast responses to mechanical stress (Fig. 8). Additionally, under mechanical loading condition, intracellular chloride ion concentration was increased and calcium signal was inhibited. Mechanical unloading, on the other hand, reduces intracellular chloride ion concentration and promotes calcium signal. Intracellular Cl^−^ levels are required for the maintenance of extracellular acidification. Ano1 knockout or Ca^2+^ binding sites mutants of Ano1 in osteoclast did not respond to mechanical loading or unloading. These findings suggest that Ano1 functions a mechanical sensor in osteoclast through its calcium regulated channel activities.

Over the past decade, osteoblasts and osteocytes were known to be the main responder to mechanical stimulation in bone tissue. The change in osteoclast function under different mechanical stimuli has been considered to be largely attributed to osteoblasts and osteocytes. Mechanical loading increases the function of osteoblasts and osteocytes^8,32,33^. Osteoclastogenesis and osteoclast function are regulated by the RANKL secretion from osteoblasts and osteocytes^34,35^. However, under the conditions of microgravity and simulated microgravity, osteoblast function is suppressed, bone mass and the mineral content are reduced, the cortical and trabecular microstructures of bone are impaired^32,36^. Yet, osteoclast function still increases under bone unloading conditions, both on Earth and in space. This raises the question of which intrinsic factors enable osteoclasts to be sensitive to mechanical stimuli, independent of changes in osteoblasts or osteocytes. Ion channels are multimeric pore-forming proteins located in the plasma membrane. They have the ability to open and close in response to different mechanical signals^37–42^. There is evidence that osteoclast differentiation can be suppressed by FSS through morphological changes and alterations in gene expression. Previous in vitro study showed that there are two types of mechanical transduction channels in response to FSS stimulation during osteoclast differentiation. Specifically, the type 1A transmembrane protein stromal interacting molecule 1 (STIM1) mediates FSS-induced [Ca^2+^]_i_ oscillation during the early stage of osteoclast differentiation, while transient receptor potential (TRP) family member TRPV4 plays an important role in FSS-induced Ca^2+^ flux and [Ca2+]_i_ oscillation in the late stage of osteoclast differentiation^43^. In our study, we found that FSS and HG stimulation inhibit Ano1 expression and channel activity by increasing intracellular [Cl^−^]_i_ and inhibiting Ca^2+^ activating downstream pathway in osteoclast. In contrast, MG stimulation promote Ano1 expression and channel activity by reducing intracellular [Cl^−^]_i_ and activating Ca^2+^ activating downstream pathway.

Recently, the cryo-EM structure of Ano1 was resolved, which revealed its structural homology to mechanosensitive channels such as OSCA1.2 and other OSCA family members^41^. They have similar dimeric architectures, transmembrane domain structures and pore locations^26^. These results suggest a potential role of Ano1 in mechanosensation. Here, we found that the response of osteoclast to mechanical stimulation is Ano1-dependent. Ano1 levels and activity underline the effect of mechanical stimulation on osteoclast. Moreover, the expression of Ano1 is modulated by different mechanical stimulation. Mechanical loading decreases Ano1 levels, while mechanical unloading increases Ano1 levels. *Ano1* knockout in osteoclast substantially bluntstheir sensitivity to mechanical stimulation. However, to determine Ano1 as a mechanosensor, the changes of architecture of Ano1 channels under mechanical stimulation need to be further elucidated.

Taken together, our findings established osteoclast activities are obviously affected by mechanical stress, which is accompanied by the altered Ano1 levels, intracellular Cl^−^ concentration and Ca^2+^ signaling. Osteoclast-specific Ano1 knockout mice are resistant to unloading-induced bone loss by blunting osteoclast response. These results demonstrate that Ano1 is an intrinsic factor that confers osteoclasts the ability to respond to mechanical stimulation. Ano1-specific inhibitors is a promising approach to counteract bone loss.

## Methods

### Mice

The *Ano1* floxed mice (*Ano1*^*fl/fl*^, a generous gift from Dr. Min-Sheng Zhu)^44^ were crossed with the Ctsk-Cre strain (a generous gift from Dr. Weiguo Zou)^45^ to generate *Ctsk-Cre;Ano1*^*fl/fl*^ mice. All mice analyzed were maintained on the C57BL/6 background. Animals were bred and maintained under specific pathogen free (SPF) conditions in Animal Research Building of China Astronaut Research and Training Center (12-h light, 12-h dark cycles, temperature controlled at 21 ± 2 °C with free access to food and water). All the experimental procedures were approved by the Committees of Animal Ethics and Experimental Safety of the China Astronaut Research and Training Center (Reference number: ACC-IACUC-2022-002).

### Cell isolation and culture

Mouse bone marrow-derived macrophages (BMMs) were obtained from tibias and femurs of 6 to 8-week-old male mice. Bone marrow cells were flushed and collected with complete α-minimal essential media (α-MEM, 10% fetal bovine serum [FBS], and penicillin/streptomycin). Cells were cultured with complete α-MEM medium in the presence of macrophage colony stimulating factor (M-CSF; 10 ng/ml, R&D Systems) for 1 day. Suspension cells were collected and inoculated onto a glass slide or culture flash. Cells were cultured in induced medium-complete medium with 30 ng/ml M-CSF and 50 ng/ml receptor activator of nuclear factor kB ligand (RANKL; R&D Systems). The culture medium was replaced every 2 days.

### TRAP staining

TRAP staining was performed using an acid phosphatase kit according to the manufacturer’s instructions (Sigma-Aldrich, catalog no.387). The cells cultured for a given period were washed with phosphate buffer solution (PBS) and fixed with 4% formaldehyde for 5 min at room temperature and rinsed thoroughly in PBS. The cells were incubated in TRAP staining solution at 37 °C for 0.5-1 h protect from light. Following removal of the TRAP solution, the plates were washed three times with distilled water. TRAP-positive multinucleated cells containing three or more nuclei were counted under an inverted microscope (Nikon).

The mouse tibiae were skinned and fixed in 4% paraformaldehyde for 48 h. The tibiae were decalcified with 10% EDTA for 10–15 days, then dehydrated and embedded with paraffin. 5–7 μm sections were prepared on a rotation microtome. The sections were dewaxed and incubated with TRAP staining solution at 37 ˚C for 1 h protect from light. Methyl green was used for counterstaining. Images were acquired with microscope and statistical analyses were performed with the BioquantOsteo Analysis System.

### RT-PCR and quantitative real-time PCR

Total RNA was extracted from cells or tissues using TRIzol reagent according to the manufacturer’s instructions. Reverse transcription was performed with 0.5 µg of total RNA in a total volume of 10 µl per reaction. RNA was reverse transcribed into cDNA with the PrimeScript RT Reagent Kit (Takara, RR037A) according to the manufacturer’s instructions. Quantitative Real-Time PCR (QRT-PCR) using a TB Green™ Premix Ex Taq™ II (Takara, RR820A). Gapdh was used as normalization control for mRNA. All primers were manufactured by BGI (Beijing, China). The following primers were used: Gapdh F: ACATCATCCCTGCATCCACTG, R: TCATTGAGAGCA ATGCCAGC; *NFATc1* F: ACGCTACAGCTGTTCATTGG, R: CTTTGGTGTTGGACAGGATG; *Acp5* F: GCGACCATTGTTAGCCACATACG, R: CGTTGATGTCGCACAGAGGGAT; *Mmp9* F: GCTGACTACGATAAGGACGGCA, R: GCGGCCCTCAAAGATGAACGG; *Ctsk* F: GCGTTGTTCTTATTCCGAGC, R: CAGCAGAGGTGTGTACTATG; *Ano1* F: CCCGTGCCAGTCACCTTTTT, R: TCATCTGCTTCCGTTTCCAGT.

### Western blotting

Cells were lysed in lysis buffer (RIPA buffer, 1 mM PMSF, Phosphatase inhibitor Cocktail and protease inhibitor cocktail) on ice for 15 min. Bone tissues were ground with a mortar in liquid nitrogen and were lysed in lysis buffer at 4 ˚C for 30 min. Protein fractions were collected by centrifugation at 12,000 × *g*, 4 ˚C for 10 min and then 10 mg of lysates were subjected to SDS-PAGE and transferred to nitrocellulose filter membrane (NC) membranes. The membranes were blocked with 5% skimmed milk and incubated with specific antibodies overnight. The antibody of used were listed: Rabbit anti-NFATc1 (1:1000, abclonal, A1539), rabbit anti-Ano1 (1:1000, abclonal, A10498), rabbit anti-p-CaMKIV antibody (1:1000, ImmunoWay, YP0043), rabbit anti-CaMKIV antibody (1:1000, Cell Signaling Technology, 4032), rabbit anti-Creb antibody (1:1000, Cell Signaling Technology, 9197), rabbit anti-p-Creb antibody (1:1000, Cell Signaling Technology, 9198), rabbit anti-Gapdh antibody (1:5000, Abways, AB0036).

### Fluid shear stress

Fluid flow was applied to cells in a parallel plate flow chamber using a closed flow loop. Osteoclast were seeded at a density of 4 × 10^6^ cells on 22 × 26 mm glass coverslips and cultured in α-MEM medium with RANKL and M-CSF for 3 days. Coverslips were placed in the parallel plate flow chamber and treated with 12 dyn/cm^2^ FSS 30 min/day, the apparatus was maintained at 37 ˚C throughout the duration of the experiment. The correlation between FSS and flow rate was calculated using the equation: τ = 6μQ/bh2, where **Q** is the flow rate (cm^3^/s), **μ** is the viscosity of the flow media (0.01 dynes/cm2), **h** is the height of the channel (0.05 cm), **b** is the slit width (2.5 cm), and **τ** is the wall shear stress (dyne/cm^2^). The parallel plate flow chamber has been described previously^8,46^.

### Hypergravity centrifuge

A hypergravity centrifuge was used to detect the effects of hypergravity on osteoclasts. In this study, osteoclasts were seeded at a density of 10^7^ cells on 12.5 cm^2^ cell culture flask and cultured in α-MEM medium with RANKL (50 ng/ml) and M-CSF (30 ng/ml) for 3 days. To prevent the presence of air bubbles, flasks were filled up with culture medium. The flasks were fixed carefully to the rotating panel of the hypergravity centrifuge, and rotated at a constant speed of 4 g hypergravity condition at 37 °C For the control, cells were cultured in the same manner but without clinorotation (1 g). The hypergravity centrifuge has been described previously^32^.

### Rotation-simulated microgravity

We used a 2D clinostat to simulate microgravity. 2D clinostat was designed and provided by the China Astronaut Research and Training Center (Beijing, China). Rotation causes a gravity vector that is not recognizable by cells. Therefore, the device prevents the cells from feeling gravity. The processing methods of osteoclasts followed to the description of rotation-simulated microgravity. The flasks were fixed carefully to the rotating panel of the clinostat system, and rotated at a constant speed of 30 rpm/min to simulate microgravity (0.01 g). For the control, cells were cultured in the same manner but without clinorotation (1 g). The 2D clinostat has been described previously^8,32^.

### Intracellular Cl^−^ concentration measurements

Osteoclasts were incubated with 5 mM MQAE (Aladdin Biochemical Technology Co., Ltd., China) for 30 min and then rinse five times with PBS. Cell fluorescence images were performed using a confocal laser scanning microscopy (CLSM, Leica SP5, Germany) with excitation light 350 nm and emission light 460 nm. Changes in [Cl^−^]_i_ were represented by the changes in the fluorescence intensity. The average fluorescence intensity of each cell was analyzed by Image J software.

### HS mouse model

The hindlimb-unloading procedure was achieved by tail suspension. All the male mice were maintained under standard animal housing conditions. The 3 month *Ano1*^*fl/fl*^ and *Ctsk-Cre;Ano1*^*fl/fl*^ male mice were individually caged and suspended by the tail using a strip of adhesive surgical tape attached to a chain hanging from a pulley. The mice were suspended at a 30˚ angle to the floor with only the forelimbs touching the floor, this allowed the mice to move and to access food and water freely. The mice were subjected to hindlimb unloading through tail suspension for 28 d. We injected calcein (30 mg/kg, sigma) intraperitoneally into mice 10 days and 2 days before euthanasia to label new bone formation. After euthanasia, the serum and whole bone tissues were collected. All of the experimental procedures were approved by the Committees of Animal Ethics and Experimental Safety of the China Astronaut Research and Training Center.

### Tibia axial loading

According to a protocol^30^, we loaded the left tibia of 16-week-old female *Ctsk-Cre;Ano1*^*fl/fl*^ mice and *Ano1*^*fl/fl*^ mice with +1200 με peak strain at the midshaft using an Electroforce BOSE 5100. The left tibia of each mouse was loaded for five consecutive days per week for 2 weeks (day 1–5 and day 8–12), and the load was applied in 1200 cycles with 4 Hz triangle waveform and 0.1 s rest time between each cycle. After euthanasia, bone tissues were collected at day 15.

### Micro-computed tomography (Micro-CT) analysis

The mouse femurs were skinned and fixed in 75% ethanol. For the distal femur and proximal tibia, the whole secondary spongiosa of the distal femur and proximal tibia from each mouse was scanned ex vivo using a microCT system (mCT40, SCANCO MEDICAL, Switzerland). Briefly, 80 slices of trabecular bone proximal to the distal growth plate was selected for analyzing the bone volume per tissue volume (BV/TV), trabecular number (Tb.N), trabecular thickness (Tb.Th) and trabecular spacing (Tb.Sp).

### Serum analysis

The analyses were performed according to the manufacturer’s instructions for serum concentrations of CTX-1 with mouse CTX1 ELISA Kit (Sangon Biotech, D721204). This assay employs the sandwich enzyme immunoassay technique. Add standard and sample to the microplate that have been pre-coated with anti-mouse CTX1 antibody. After incubation, add biotin-conjugated anti-mouse CTX1 antibody. It is then combined with HRP-conjugated streptavidin to form an immune complex, then incubated and washed to remove unbound enzyme, and then added to the chromogenic substrate TMB to produce a blue color, and converted to the final yellow under the action of acid. Finally, the absorbance (OD) value was measured at 450 nm. The concentration of mouse CTX1 in the sample was proportional to the OD value. The concentration of mouse CTX1 in the sample can be calculated by drawing a standard curve.

### Statistics and reproducibility

Cell-based experiments were performed at least three independent replicates. Animals were randomized into different groups and at least 5 mice were used for each group. The data are presented as mean ± s.e.m. Student’s *t* test was used for statistical evaluations of two group comparisons. Statistical analysis with more than two groups was performed with one-way analysis of variance (ANOVA). All statistical analyses were performed with Prism software (Graphpad prism for windows, version 8.0). Differences were considered significant at ^*^P < 0.05, ^**^P < 0.01, ^***^P < 0.001.

## Supplementary information

Supplementary Figures

Description of Additional Supplementary Data

Supplementary Data

reporting-summary

## Data Availability

The data generated or analyzed during this study are provided in the main paper (Figs. 1–8) and supplementary figures (Supplementary Figs. 1–4). The original gel and blot images are shown in the Supplementary Figs. 5–8. The source data for the graphs and images can be found in the Supplementary Excel files (Supplementary Data). All resources are also available from the authors upon reasonable request.
