# Supplementary material for: Mechanical stimulation controls osteoclast function through the regulation of Ca2+-activated Cl− channel Anoctamin 1
Source: Commun Biol. 2023 Apr 13;6:407. doi: 10.1038/s42003-023-04806-1 (PMC10102170; doi:10.1038/s42003-023-04806-1)

Supplemetary Fig.1

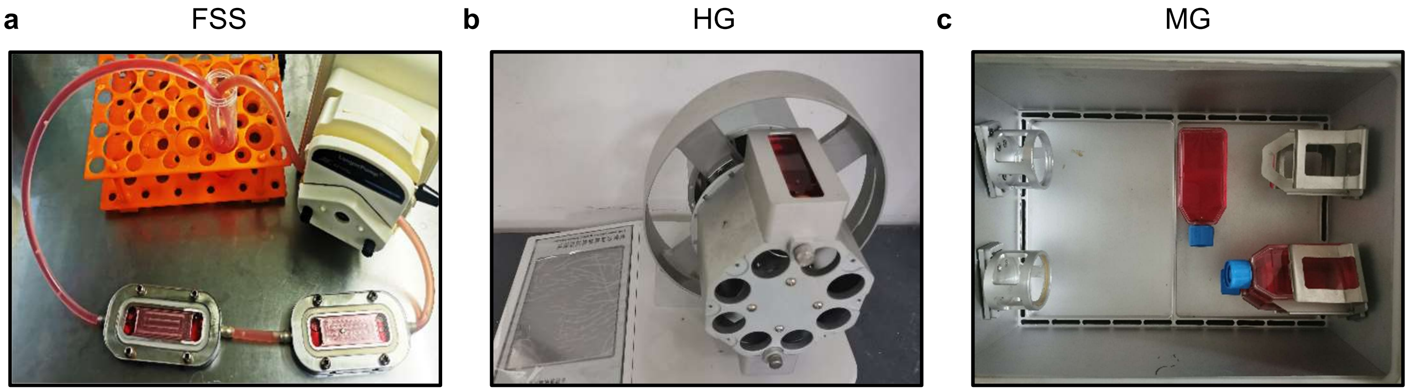

Supplemetary Fig.1 Different mechanical stimulation models

(**a**) The image of fluid shear stress system. (**b**) The image of hypergravity centrifuge. (**c**) The image of clinostat device that used to simulate microgravity.

Supplemetary Fig.2

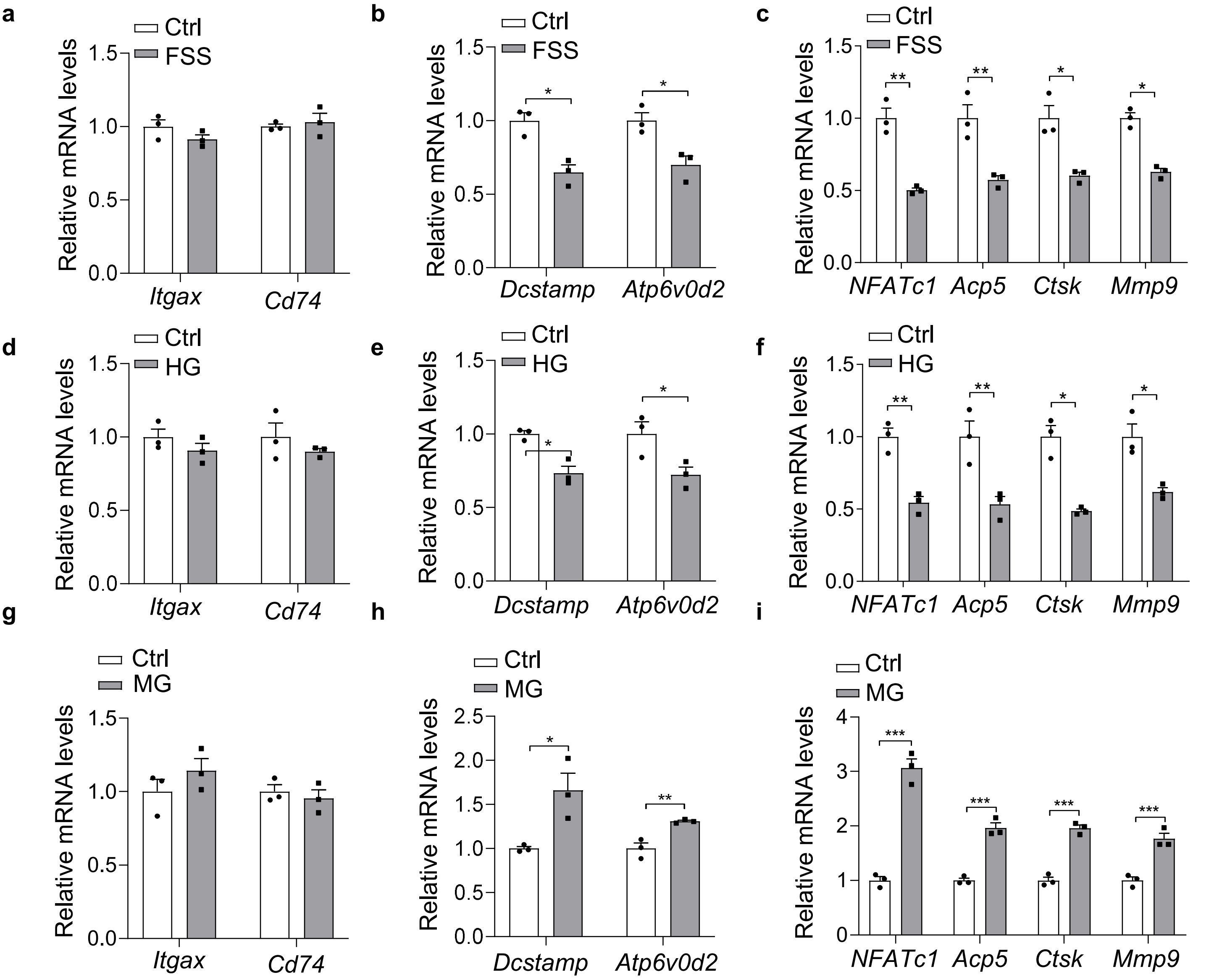

Supplementary Fig. 2 Mechanical stimulation affects osteoclast differentiation

(**a**) QRT-PCR analysis of *Itgax* and *Cd74* mRNA levels in bone marrow-derived macrophages (BMMs) induced by RANKL and treated with control (Ctrl) or fluid shear stress (FSS, 12 dyn/cm^2, 30 min/day) for 1 day. (**b**) QRT-PCR analysis of *Dcstamp* and *Atp6v0d2* mRNA levels in BMMs induced by RANKL and treated with Ctrl or FSS for 3 day. (**c**) QRT-PCR analysis of *NFATc1*, *Acp5*, *Ctsk* and *Mmp9* mRNA levels in BMMs induced by RANKL and treated with Ctrl or FSS for 5 day. (**d**) QRT-PCR analysis of *Itgax* and *Cd74* mRNA levels in BMMs induced by RANKL and treated with normal gravity (Ctrl, 1 g) or hypergravity (HG, 4g) for 1 day. (**e**) QRT-PCR analysis of *Dcstamp* and *Atp6v0d2* mRNA levels in BMMs induced by RANKL and treated with Ctrl or HG for 3 day. (**f**) QRT-PCR analysis of *NFATc1*, *Acp5*, *Ctsk* and *Mmp9* mRNA levels in BMMs induced by RANKL and treated with Ctrl or HG for 5 day. (**g**) QRT-PCR analysis of *Itgax* and *Cd74* mRNA levels in BMMs induced by RANKL and treated with Ctrl or simulated microgravity (MG) for 1 day. (**h**) QRT-PCR analysis of *Dcstamp* and *Atp6v0d2* mRNA levels in BMMs induced by RANKL and treated with Ctrl or MG for 3 day. (**i**) QRT-PCR analysis of *NFATc1*, *Acp5*, *Ctsk* and *Mmp9* mRNA levels in BMMs induced by RANKL and treated Ctrl or MG for 5 day. All data are the mean ± s.e.m. from three independent experiments. Statistical analysis with more than two groups was performed with two-way analysis of variance (ANOVA) with Šídák post-hoc test to determine group differences. $^*p < 0.05$, $^{**}p < 0.01$, $^{***}p < 0.001$.

Supplemetary Fig.3

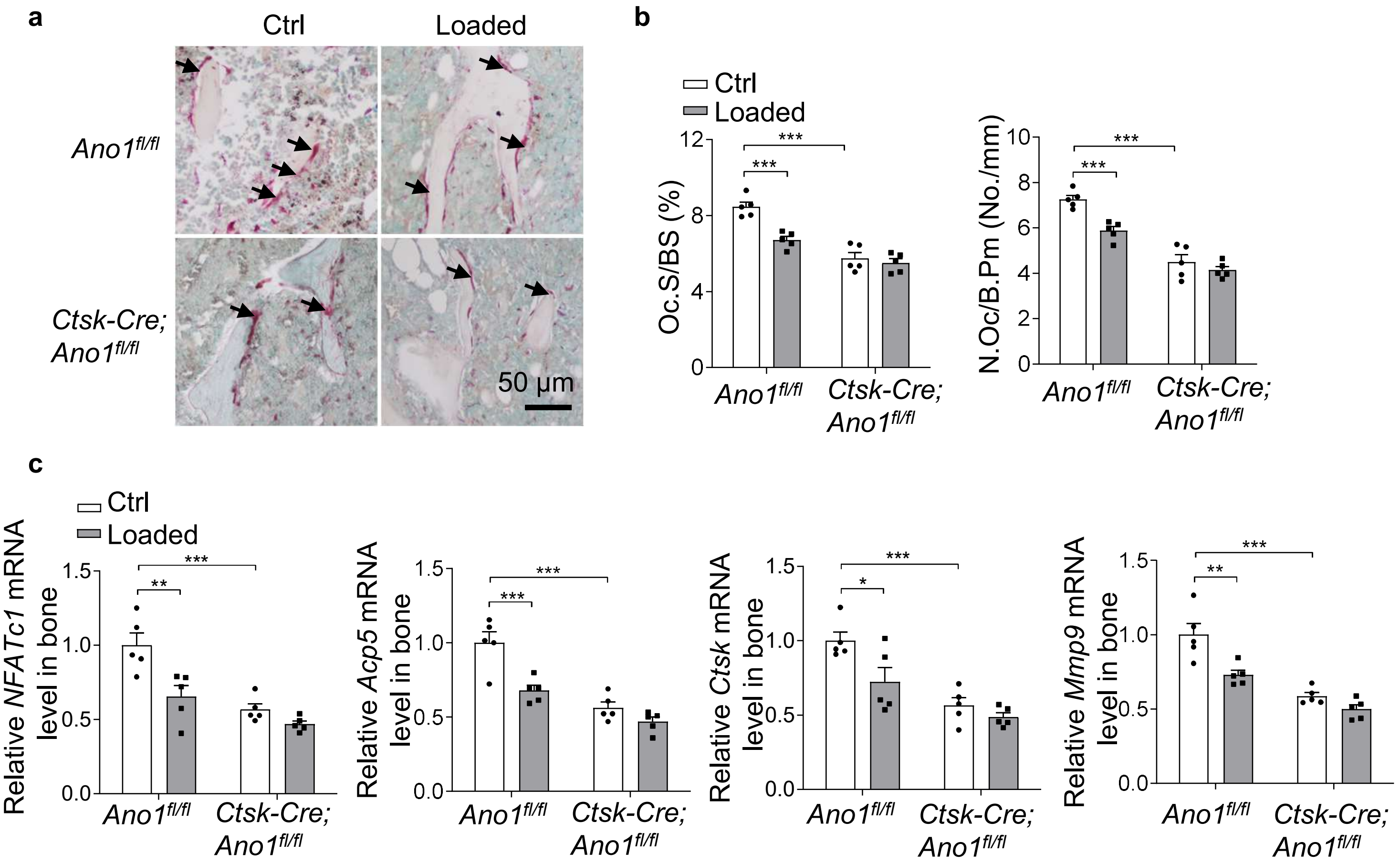

Supplementary Fig. 3 *In vivo*, Ano1 knock out attenuates the effect of mechanical load on osteoclast activity

(**a**) Representative images of TRAP staining of the tibia from the groups of mice indicated. Scale bar, 50 μm. (**b**) Histomorphometry analysis of the images for number of osteoclasts per bone perimeter (N.Oc/B.Pm) and osteoclast surface per bone surface (Oc.S/BS) of the tibia from 4-month-old *Ano1$^{fl/fl}$* (n = 5) and *Ctsk-Cre;Ano1$^{fl/fl}$* (n = 5) mice with loaded or Ctrl treatment. (**c**) QRT-PCR analysis of *NFATc1*, *Acp5*, *Ctsk* and *Mmp9* mRNA levels in bone tissues collected from the groups of mice indicated. n = 5 for each group. Statistical analysis with more than two groups was performed with two-way analysis of variance (ANOVA) with Šídák post-hoc test to determine group differences. *p < 0.05, **p < 0.01, ***p< 0.001.

Supplemetary Fig.4

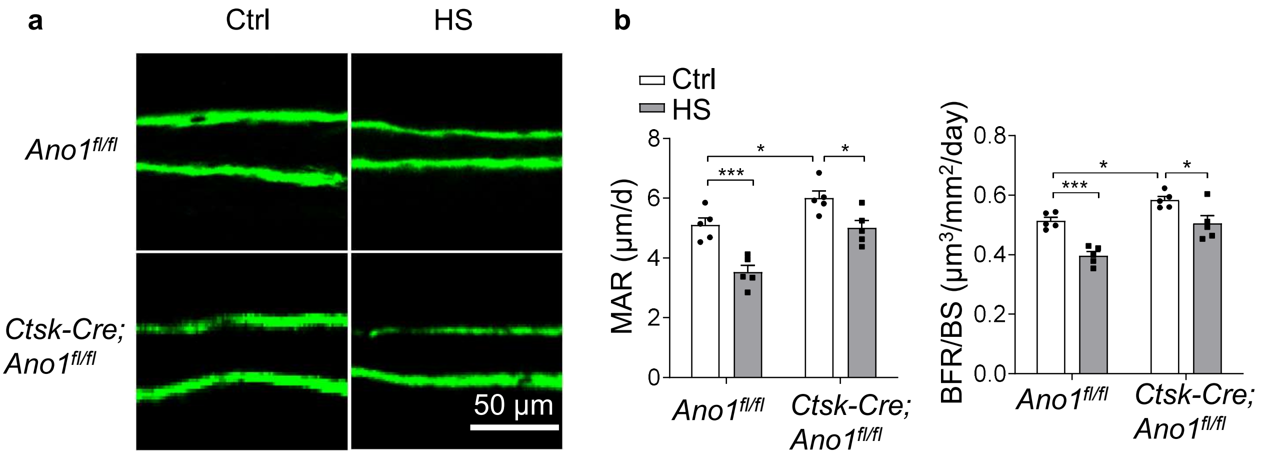

Supplementary Fig.4 Osteoclast specific Ano1 knock out attenuates the inhibition of unloading on bone formation

(**a**) Representative images showing new bone formation assessed by double calcein labeling in the groups of mice indicated. Scale bar, 50 μm. (**b**) Mineral apposition rate (MAR) and bone formation rate per bone surface (BFR/BS) of the tibia from the groups of mice indicated. 4-month-old female *Ano1$^{fl/fl}$* (n = 5) and *Ctsk-Cre;Ano1$^{fl/fl}$* (n = 5) mice treatment with HS or Ctrl for 28 days. Statistical analysis with more than two groups was performed with two-way analysis of variance (ANOVA) with Šídák post-hoc test to determine group differences. **p* < 0.05, ****p*< 0.001.

Supplemetary Fig.5

Fig.1a

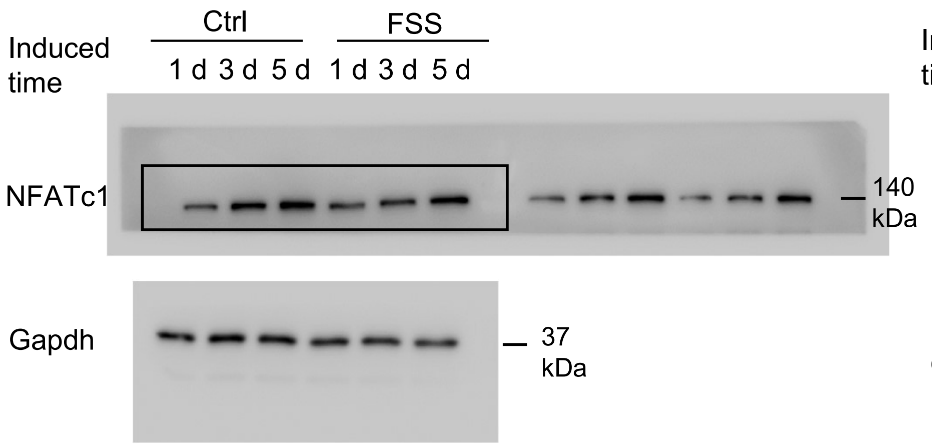

Fig.1c

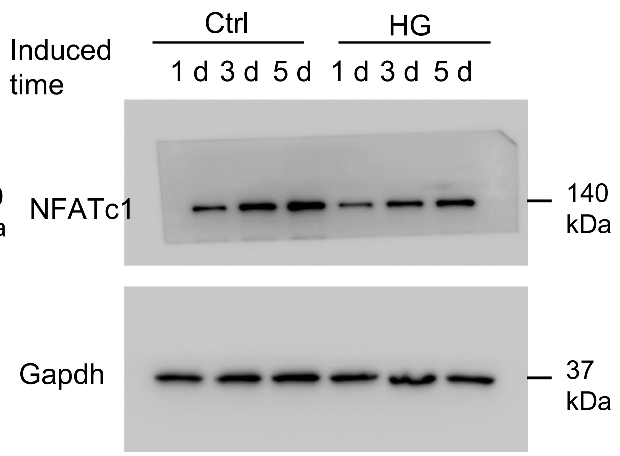

Fig.1e

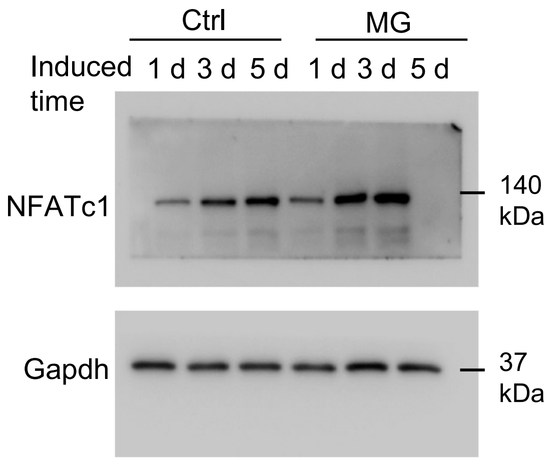

Fig.1h

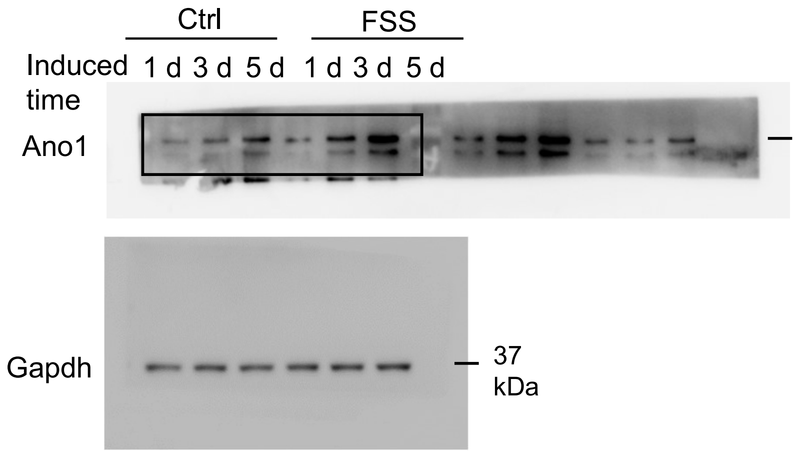

Fig.1j

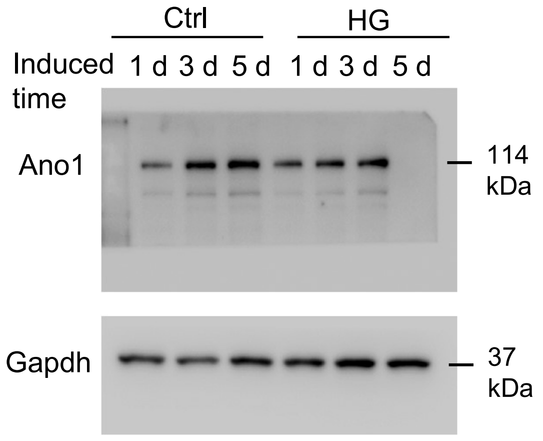

Fig.1l

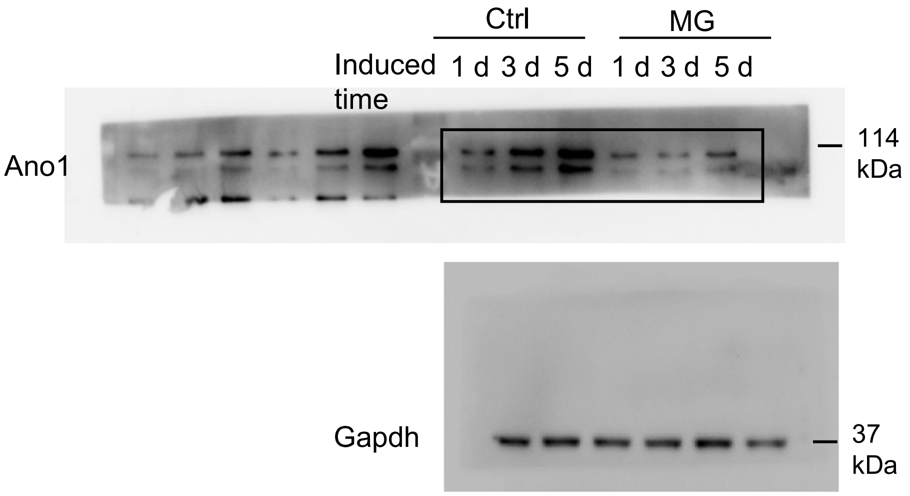

Supplemetary Fig.6

Fig.2b

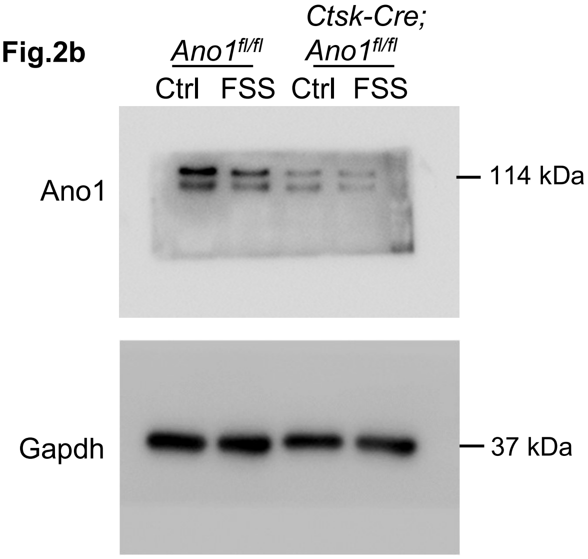

Fig.3b

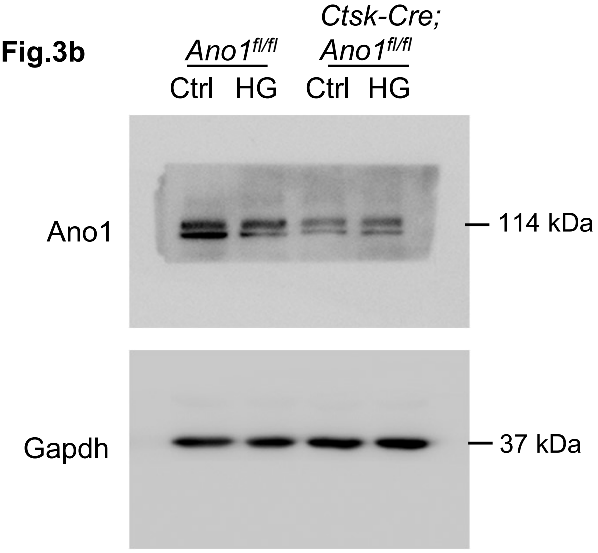

Fig.4b

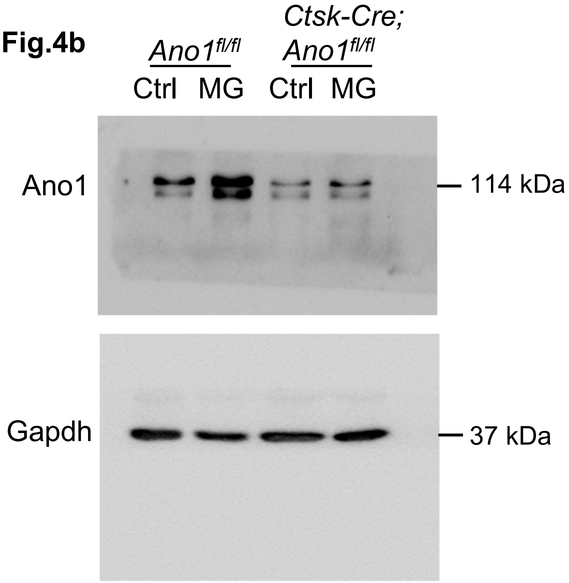

Supplemetary Fig.7

Fig.6a

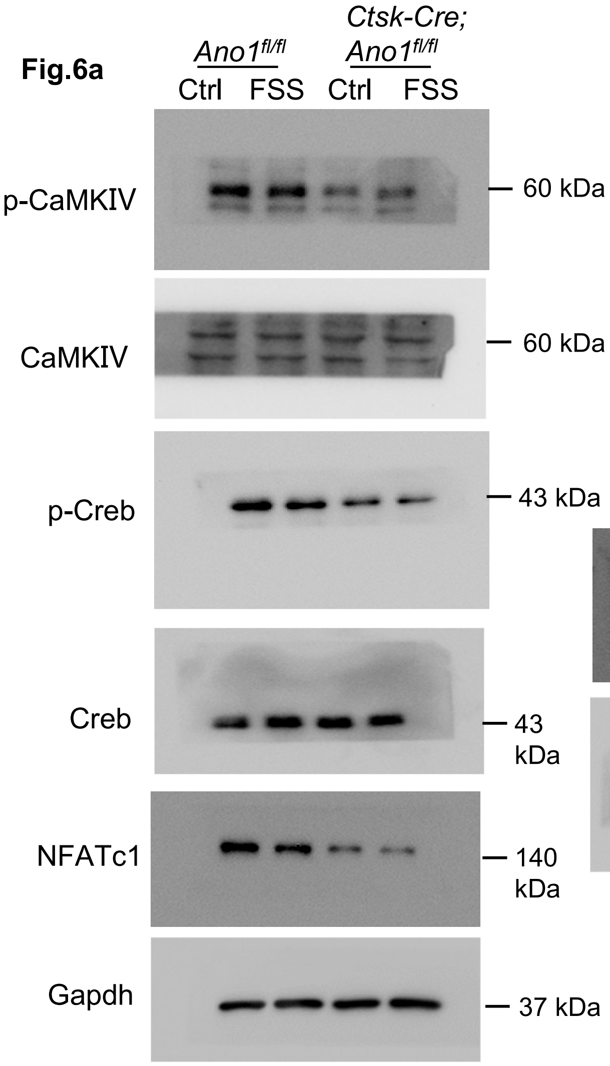

Fig.6e

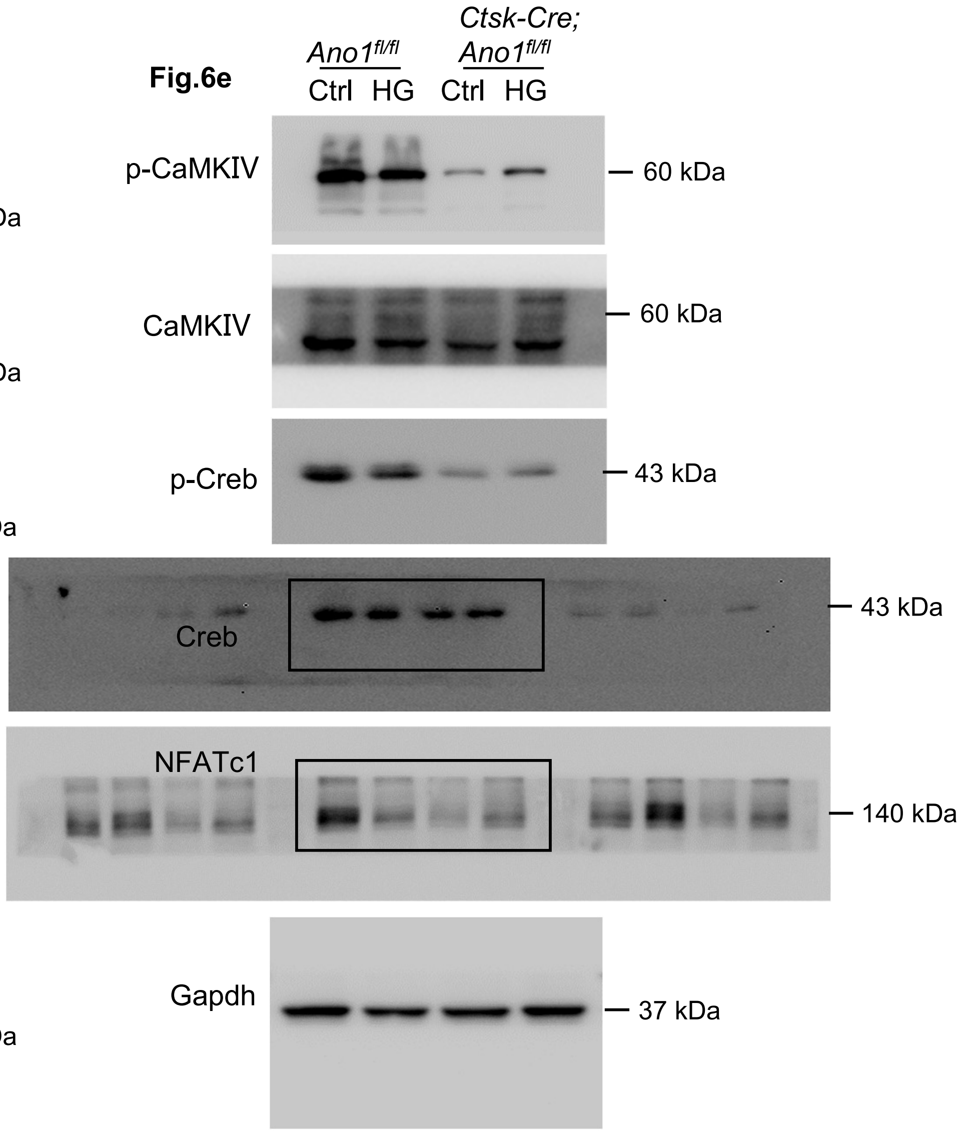

Fig.6i

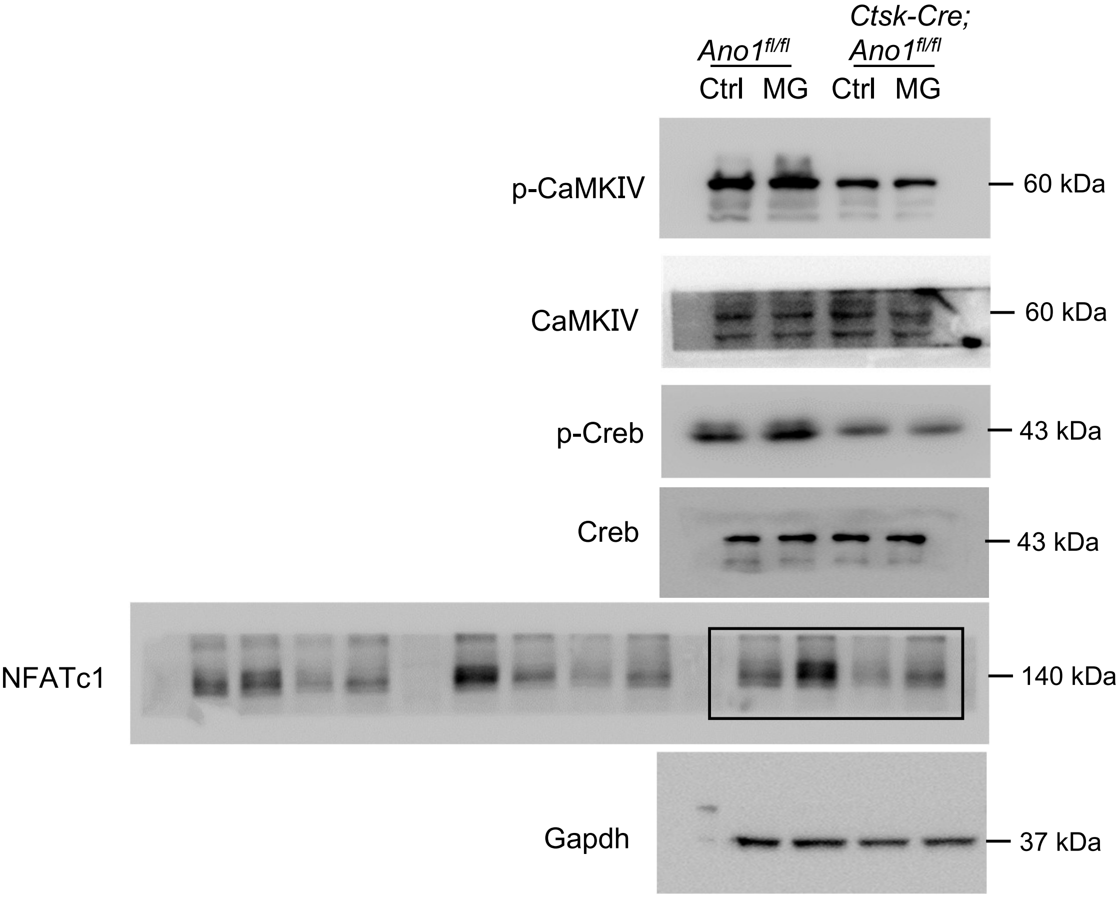

Supplemetary Fig.8

Fig.7b

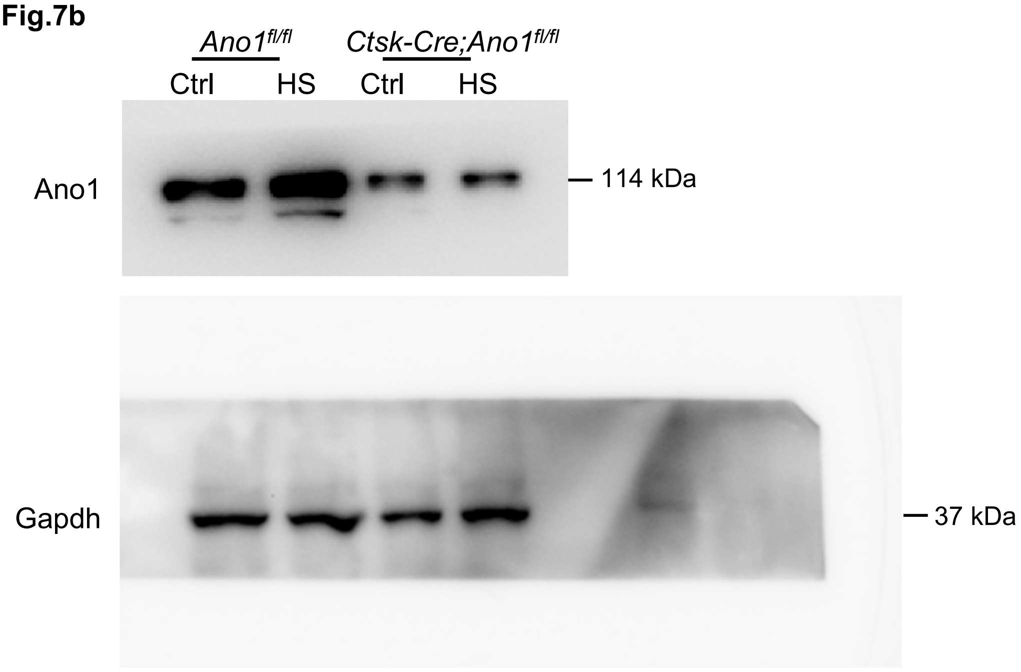

Supplement: Supplementary file 1 — Supplementary Figures [file 42003_2023_4806_MOESM1_ESM.pdf]
